# CD9 Is a Very Helpful Marker for Discriminating AML-M3 from HLA-DR-Negative Non-M3 AML

**DOI:** 10.4274/tjh.galenos.2020.2020.0110

**Published:** 2020-11-19

**Authors:** Esmaeil Shahabi Satlsar, Mohammad Mosleh, Mahdieh Mehrpouri

**Affiliations:** 1 Guilan University of Medical Sciences,School of Paramedical Sciences, Clinical Laboratory Sciences Department, Rasht, Iran; 2 Thakhte Tavous Pathobiology Laboratory, Flow Cytometry Department, Tehran, Iran; 3 Shahid Beheshti University of Medical Sciences, Hematology and Blood Banking Department, School of Paramedical Sciences, Tehran, Iran; 4 Alborz University of Medical Sciences, School of Paramedical Sciences Clinical Laboratory Sciences Department, Karaj, Iran

**Keywords:** AML-M3, CD9, Flow cytometry, HLA-DR-negative AML

## To the Editor,

CD9 is a cell-surface marker whose carcinogenic properties have been proven in several solid tumors. Previous studies reported that the blast cells of both B-cell acute lymphoblastic leukemia (ALL) and acute myeloid leukemia (AML), as well as normal B-cell precursors (hematogones), express CD9 [1,2,3]. Acute promyelocytic leukemia (APL) is a highly aggressive type of AML with an increased risk of death due to hemorrhage, and flow cytometry provides an accessible and useful tool for the rapid diagnosis of APL. Promyelocytes in APL are characterized by negative expression of HLA-DR, CD11b, and CD34 and they are often positive for cMPO (bright), CD13, CD33, CD34 (dim), CD64, and CD117 (dim to moderate). Another subtype of AML known as HLA-DR-negative non-M3 AML also lacks the expression of HLA-DR and CD34 [4,5]. Differential diagnosis of APL from HLA-DR-negative non-M3 AML cannot be based on morphology and lack of HLA-DR antigen expression; rather, it requires molecular confirmation of PML-RARA using cytogenetic analysis, which leads to delayed diagnosis. In our previous study evaluating the expression of CD9 in AML cases [6], we showed differences in the expression of CD9 between blasts of APL and HLA-DR-negative non-M3 AML. With respect to the interesting results of the previous study, we decided to continue evaluating this marker in patients with APL and HLA-DR-negative non-M3 AML. Accordingly, we evaluated CD9 expression in 101 patients with APL and 94 patients with HLA-DR-negative non-APL using flow cytometry; moreover, molecular evaluation for PML-RARA was performed for all studied patients for confirming the suspected diagnosis of APL.

**Figure 1:**
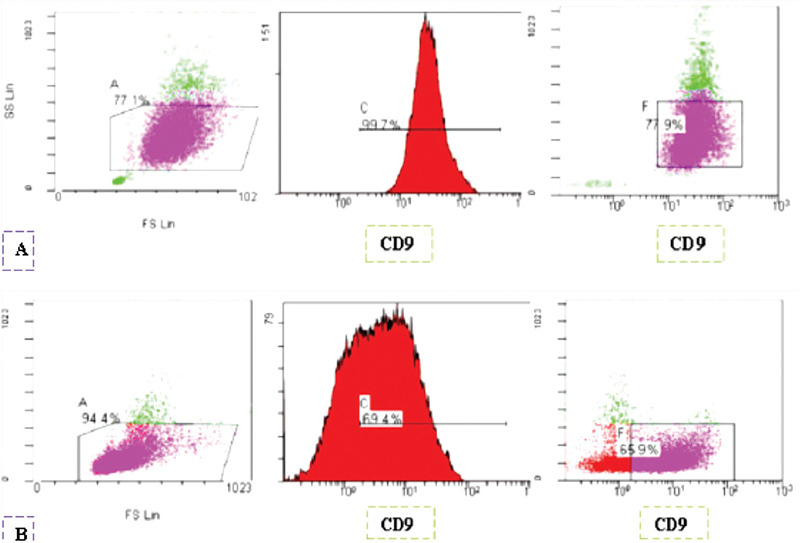
Differences in CD9 expression patterns in APL and HLA-DR-negative non-APL: A) APL blasts show homogeneous and moderate to bright expression, B) HLA-DR-negative non-APL cases have dim to moderate and heterogeneous expression.

Flow cytometric analysis was performed using a Beckman Coulter Cytomics FC 500 flow cytometer with MXP software. Bone marrow and/or peripheral blood samples were taken from the patients between March 2015 and January 2020. The following conjugated monoclonal antibodies were used in four-color combinations: CD9 (FITC-Coulter), HLA-DR (PE-Dako), HLA-DR (PE-Immunostep), CD13 (Percp/Cy5-BD), CD33 (APC-Coulter), CD64 (PE-Dako), CD117 (PE-Coulter), CD34 (PE-BD), and CD45 (Percp-Immunostep). Resulting data showed that both APL patients and HLA-DR-negative non-APL patients expressed the CD9 marker, although each group had a distinct pattern of expression. Consistent with that finding, our data revealed that all APL patients (100%) showed homogeneous and moderate or bright expression of CD9, similar to the pattern of CD33 expression. Conversely, in HLA-DR-negative non-APL patients, CD9 expression was detected in 59 of 94 (62.7%) of cases, with a heterogeneous pattern and dim to moderate expression, which is a completely different pattern from that seen in cases of APL (Figure 1).

The timely diagnosis of APL continues to be challenging despite advancements in medical diagnostics in many countries of the world. Furthermore, the abnormal morphology of promyelocytes, especially in the hypogranular variants [7], and the expression of CD11b can lead to diagnostic errors in some cases.

The most straightforward explanation of our results is that expression patterns of CD9, along with other myeloid markers such as CD13, CD33, and CD64, can be helpful in the precise differential diagnosis of patients with APL from patients with HLA-DR-negative non-APL.
